# Production of Saffron Apocarotenoids in *Nicotiana benthamiana* Plants Genome-Edited to Accumulate Zeaxanthin Precursor

**DOI:** 10.3390/metabo13060729

**Published:** 2023-06-06

**Authors:** Olivia Costantina Demurtas, Maria Sulli, Paola Ferrante, Paola Mini, Maricarmen Martí, Verónica Aragonés, José-Antonio Daròs, Giovanni Giuliano

**Affiliations:** 1 Biotechnology and Agro-Industry Division, ENEA, Italian National Agency for New Technologies, Energy and Sustainable Economic Development, Casaccia Research Center, 00123 Rome, Italy; maria.sulli@enea.it (M.S.); paola.ferrante@enea.it (P.F.); paola.mini@enea.it (P.M.); 2 Instituto de Biología Molecular y Celular de Plantas, Consejo Superior de Investigaciones Científicas-Universitat Politècnica de València, 46022 Valencia, Spain; mamarbo5@alumni.upv.es (M.M.); varagoni@ibmcp.upv.es (V.A.); jadaros@ibmcp.upv.es (J.-A.D.)

**Keywords:** zeaxanthin, *Nicotiana benthamiana*, crocins, picrocrocin, agroinfiltration, agroinoculation, new plant breeding techniques (NPBTs), vacuole

## Abstract

Crocins are glycosylated apocarotenoids with strong coloring power and anti-oxidant, anticancer, and neuro-protective properties. We previously dissected the saffron crocin biosynthesis pathway, and demonstrated that the CsCCD2 enzyme, catalyzing the carotenoid cleavage step, shows a strong preference for the xanthophyll zeaxanthin in vitro and in bacterio. In order to investigate substrate specificity in planta and to establish a plant-based bio-factory system for crocin production, we compared wild-type *Nicotiana benthamiana* plants, accumulating various xanthophylls together with α- and β-carotene, with genome-edited lines, in which all the xanthophylls normally accumulated in leaves were replaced by a single xanthophyll, zeaxanthin. These plants were used as chassis for the production in leaves of saffron apocarotenoids (crocins, picrocrocin) using two transient expression methods to overexpress *CsCCD2*: agroinfiltration and inoculation with a viral vector derived from tobacco etch virus (TEV). The results indicated the superior performance of the zeaxanthin-accumulating line and of the use of the viral vector to express *CsCCD2*. The results also suggested a relaxed substrate specificity of CsCCD2 in planta, cleaving additional carotenoid substrates.

## 1. Introduction

**Figure 1:**
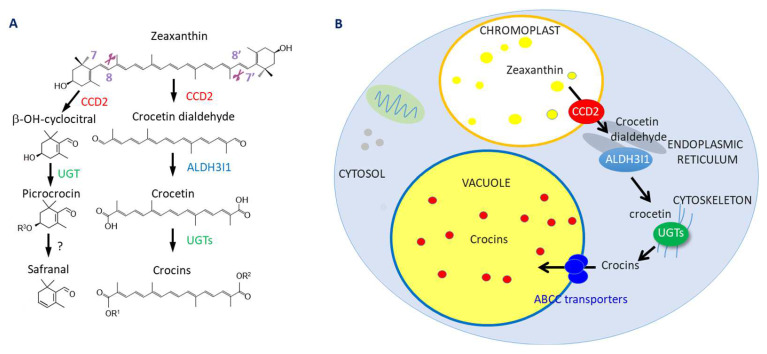
(**A**) Crocin-picrocrocin-safranal biosynthesis pathway in *C. sativus*, and (**B**) its subcellular compartmentation. R^1^ and R^2^ in panel A are sugars or H atoms, as previously described [4], R^3^ is a glucose, as previously described [6].

Crocins are high-value hydrophilic pigments that accumulate at high concentrations in the stigmas and/or tepals of different *Crocus* (Iridaceae) flowers and reach particularly high levels in the stigmas of *Crocus sativus* (saffron), the most expensive spice in the world [1,2]. We and others have previously dissected the biosynthetic pathway in *Crocus* stigmas (Figure 1). The first dedicated step is the 7,8/7′,8′ cleavage of zeaxanthin by carotenoid cleavage dioxygenase 2 (CsCCD2) [3], followed by dehydrogenation of crocetin dialdehyde by CsALDH3I1 and primary glycosylation by CsUGT74AD1 [4]. Secondary glycosylation is exerted by CsUGT91P3 [5]. The cleavage of zeaxanthin at the 7,8/7′,8′ double bonds also produces OH-β-cyclocitral [3] that is glycosylated to picrocrocin, by the activity of CsUGT709G1 [6]. Picrocrocin and the derived volatile apocarotenoid, named safranal, are responsible for the characteristic saffron taste and aroma, respectively.

The crocin pathway is highly compartmentalized (Figure 1): CsCCD2 is localized into the plastid, CsALDH3I1 in the endoplasmic reticulum, and CsUGT74AD1 in cytoskeleton-like cytoplasmic structures [4]. Crocins are transported into vacuoles by ABCC-type transporters [7].

The crocin biosynthesis pathway has also been elucidated in *Gardenia jasminoides* [8] and partially in *Buddleja davidii* [9,10] and *Bixa orellana* [11]. In these cases, a CCD4 is involved in the carotenoid cleavage step.

Different crocin-synthesizing CCDs show different substrate specificities in bacterio and/or in vitro: the *Crocus* and *Buddleja* CCDs cleave preferentially zeaxanthin [3,9], while the *Gardenia* and *Bixa* enzymes have a more relaxed substrate specificity, cleaving lycopene, beta-carotene, and zeaxanthin [8,11].

Interestingly, expression in planta of the above CCDs is sufficient for crocin accumulation, suggesting that the ALDH and UGT activities are endogenously expressed in plant tissues. Heterologous expression systems include maize endosperm [3], rice and citrus calli [12,13], *Nicotiana benthamiana* leaves [7,11,14], transplastomic tobacco leaves [11], *N. tabacum* and *N. glauca* leaves [15,16], and transgenic tomato fruits [11,17]. Since many of these tissues contain a variety of different carotenoids, but very low to null levels of zeaxanthin, the question arises about which are the in planta carotenoid substrates of CCDs with narrow substrate specificity, such as CsCCD2. This question is particularly relevant to develop a bio-factory system to produce the valuable crocins in heterologous plants. To answer this question, we generated genome-edited plants of *N. benthamiana* in which the normal leaf xanthophyll complement (lutein, violaxanthin, antheraxanthin, and neoxanthin) was replaced by zeaxanthin, a xanthophyll that is normally accumulated in leaves only under high-light conditions (Sulli et al., unpublished). In this report we used both *Agrobacterium tumefaciens*-mediated infiltration, and inoculation with a custom-built tobacco etch virus (TEV; genus *Potyvirus*; family *Potyviridae*), to overexpress the saffron CsCCD2 dioxygenase, which catalyzes the first dedicated step in crocin biosynthesis. Results suggested that depending on the overexpression method used, CsCCD2 showed relaxed substrate specificity in planta, also cleaving lutein and, to a lesser extent, violaxanthin and β-carotene. Remarkably, we obtained a maximum apocarotenoid and crocin yields (1129.6 and 883.7 µg g^−1^ dry weight (DW), respectively) in genome-edited plants with the highest levels of zeaxanthin, in which *CsCCD2* was expressed with the viral vector.

## 2. Materials and Methods

### 2.1. Plant Material

*N. benthamiana* laboratory isolate (LAB) plants [18], edited in the two homologous *Lycopene ε-cyclase* (*LCYe*) and two *Zeaxanthin epoxidase* (*ZEP*) genes (*LCYe1-LCYe2*: *Nbv6.1trP33255-Nbv6.1trP19906*; *ZEP1-ZEP2*: *Nbv6.1trP61527-Nbv6.1trP32819*) [19] (Sulli et al., unpublished) were utilized. Two different high zeaxanthin (HZ) lines were utilized: HZ-9 (*lcye1lcye2ZEP1zep2*) in which only one of the two *ZEP* genes was inactivated, and HZ-11 (*lcye1lcye2zep1zep2*) in which both *ZEP* genes were inactivated.

### 2.2. Agroinfiltration

Wild-type (Wt) and HZ plants were grown in soil for 5–6 weeks in a growth chamber with controlled temperature (22 °C) and photoperiod (16 h light/8 h dark, 150 µE m^−2^ s^−1^, given from five Lumilux Cool Daylight L 58W/865 and one Fluora L 58W/77 fluorescent lamps, Osram, Germany). Agroinfiltration was performed by inoculating sub-apical leaves of the second and third whorls, as previously described [7,11]. For each plant, the 4 sub-apical leaves of each agroinfiltrated plant were harvested at 5 days post inoculation (dpi), pooled, and frozen in liquid nitrogen. Three independent experiments were performed. *N. benthamiana* Wt and HZ lines were co-infiltrated with *A. tumefaciens* C58C1 cells containing pBI121:CsCCD2 [7] or pBI121 [20] as negative control, and the RK19 silencing suppressor [21].

### 2.3. Virus Inoculation

Plasmid pGTEV:CsCCD2 was built using standard molecular biology techniques with the corresponding restriction enzymes (Thermo Fisher Scientific, Waltham, MA, US) and T4 DNA ligase (Thermo Fisher Scientific, Waltham, MA, US). The Eco81I-ApaI DNA fragment of pGTEVΔNIb-CsCCD2L [14] was replaced by the same restriction fragment from pGTEVa [22]. This plasmid contains a full-length TEV infectious clone including the viral NIb cistron. pGTEV-eGFP is a pGTEVa derivative that contains the cDNA of the enhanced GFP (AAB08060) inserted between the viral NIb and CP cistrons [23]. The GFP cDNA is flanked by sequences corresponding to artificial NIaPro cleavage sites that mediate the release of the recombinant protein from the viral polyprotein [23]. The full nucleotide sequences of recombinant viruses TEV-CsCCD2L and TEV-eGFP are shown in Figure S1. Empty plasmid pG35Z was used to mock-inoculate plants [24]. *A. tumefaciens* C58C1, harboring the helper plasmid pCLEAN-S48 [25], were transformed with plasmids pGTEV:CsCCD2L, pGTEV:GFP, and pG53Z, and selected in plates containing 50 µg mL^−1^ rifampicin, 50 µg mL^−1^ kanamycin, and 7.5 µg mL^−1^ tetracycline. Selected colonies were grown overnight at 28 °C in Luria-Bertani (LB) liquid media containing 50 µg mL^−1^ kanamycin. Cells were brought to optical density (600 nm) of 0.5 in 10 mM MES-NaOH, pH 5.6, 10 mM MgCl_2_, and 150 μM acetosyringone, and *Vir* genes induced for 2 h at 28 °C. Using a needleless syringe, cultures were infiltrated in the abaxial side of two leaves of five-week-old *N. benthamiana* Wt and genome-edited (HZ-9 and HZ-11) plants. After agroinoculation, plants were cultured in a growth chamber at 25 °C under a 12 h day–night photoperiod with an average photon flux density of 240 µE m^−2^ s^−1^. Symptomatic systemic leaves were collected at 10 dpi and processed for metabolite analysis.

### 2.4. Extraction and Analysis of Carotenoids

Carotenoids were extracted from 10 mg of ground, lyophilized leaf tissue under dim laboratory light conditions (<5 µE m^−2^ s^−1^) during the entire extraction procedure. Samples were homogenized (20 min, 30 Hz) with 1.8 mL 100% cold acetone spiked with 50 mg L^−1^ of internal standard (DL-α-tocopherol acetate, Sigma-Aldrich, Cat. No. 47786, St. Louis, MO, USA), using the TissueLyser MM300 (Retsch, Haan, Germany). Samples were centrifuged (15 min, 20,000× *g*, 4 °C), and supernatant was transferred in two new tubes. A quantity of 0.8 mL acetone was directly transferred into a new tube, centrifuged (15 min, 20,000× *g*, 4 °C), and 0.6 mL of the supernatant was filtered using Whatman™ Mini-UniPrep™ Amber Syringeless Filter vials (0.45 µm PTFE, Merck, Darmstadt, Germany) for LC analysis. A quantity of 0.9 mL was directed to saponification, and thus transferred to a 2 mL tube containing 100 mg of Ambersep 900 OH resin (Merck, Darmstadt, Germany), which was pre-treated with ultrapure water (LiChrosolv^®^, Merck, Darmstadt, Germany) as previously described [26]. After shaking for 1 h in the dark and centrifugation (15 min, 14,000× *g*, 4 °C), 0.6 mL of supernatant was removed and transferred into filtered Whatman™ Mini-UniPrep™ Amber Syringeless Filter vials (0.45 µm PTFE, Merck, Darmstadt, Germany) for LC analysis. Based on previous experience, this protocol results in recovery efficiencies >90%. Analytical HPLC was carried out on a LC-PhotoDiode Array (PDA) system (Accela, Thermo Fisher Scientific, Thermo Fisher Scientific, Waltham, MA, USWaltham, MA, US). LC separation was performed using a Carotenoid C30 column (100 × 3 mm^2^, 3-µm particle size) (YMC, Dinslaken, Germany) and the column temperature was 25 °C. The elution system was A, MeOH; B, MeOH/water (4:1 *v*/*v*) with 0.2% ammonium acetate; and C, tert-Butyl methyl ether (C). The gradient was 0 to 1.2 min 95% A, 5% B; 3.5 min 80% A, 5% B; 6.8 min 30% A, 5% B, 65% C; 16 min 95%, 5% B. PDA data were recorded in the 200–700 nm range [27]. Chromatographic flux after equilibration was 0.8 mL/min and total run time was 18 min, and the injection volume was 10 µL. All the solvents used were LC grade (Merck, Darmstadt, Germany). For quantification, carotenoid peaks were integrated at their individual λmax and DL-α-tocopherol acetate at 285 nm as previously described [28]. Data were then normalized on the peak area of the internal standard and on the dry weight of leaf tissue used for the extraction [29,30,31,32]. The limits of detection and quantitation for individual carotenoid peaks were 1500 and 2000 μAU, respectively.

### 2.5. Extraction and Analysis of Crocins

Semi-polar metabolites were extracted as previously described with some modifications [11]. Briefly, 10 mg of powder was ground and resuspended in 750 µL of 75% (*v*/*v*) cold methanol spiked with 0.5 µg mL^−1^ formononetin (Sigma–Aldrich, Cat. No. 47752-25MG-F, St. Louis, MO, USA) with vigorous agitation for 30 min at 25 °C. Samples were then centrifuged at 20,000× *g* for 20 min; the supernatant was collected, filtered with HPLC PTFE filter tubes (0.22 µm pore size), and subjected to high performance liquid chromatography-photodiode array detection-high resolution mass spectrometry (LC–PDA–HRMS) analysis using a Q-Exactive mass spectrometer (Thermo Fisher Scientific, Waltham, MA, USA) as previously described [4]. Based on previous experience, this protocol results in recovery efficiencies >90%. Briefly, the separation was performed by injecting 5 µL of sample on a C18 Luna reverse-phase column (100 × 2.1 mm^2^, 2.5 µm; Phenomenex, Torrance, CA, USA) using as mobile phases water + 0.1% formic acid (A) and acetonitrile + 0.1% formic acid (B) at a total flow rate of 250 µL/min. The separation was developed using 5% B for 0.5 min, followed by a 24 min linear gradient to 75% B. The ionization of polar and semi-polar apocarotenoids was performed using a heated electrospray ionization (HESI, Thermo Fisher Scientific, Waltham, MA, USA) source with nitrogen used as a sheath and auxiliary gas, set to 45 and 30 units, respectively. The vaporizer temperature was 270 °C, the capillary temperature was 30 °C, the discharge current was set to 5 μA, and the S-lens RF level was set at 50. The acquisition was performed in the mass range 110–1600 *m*/*z* both in positive and in negative ion modes with the following parameters: resolution 70,000, microscan 1, AGC target 1 × 10^6^, maximum injection time 50. All the solvents used were LC-MS grade (Merck, Darmstadt, Germany). PDA data were recorded in the 200–700 nm range. Crocins, crocetin, picrocrocin, and OH-β-cyclocitral were identified in positive ionization mode in LC-MS and LC-MS/MS chromatograms and quantified in PDA chromatograms as follows: crocin and crocetin peak areas were integrated at their λmax = 443 nm, picrocrocin and OH-β-cyclocitral at λmax = 257 nm, and the internal standard formononetin at λmax = 256 nm; the areas were quantified considering the specific extinction coefficients (ε1%) of each compound, using the ε1% values reported for crocins and crocetins [33], for picrocrocin and its precursor OH-β-cyclocitral [34], and for the internal standard (formononetin) [35]. Data were then normalized on the peak area of the internal standard and on the dry weight of leaf tissue used for the extraction. The limits of detection and quantitation for individual crocin/picrocrocin peaks were 1000 and 1500 μAU, respectively.

### 2.6. Statistical Analyses

The Past software (version 3.11) [36] was used to identify carotenoids/apocarotenoids showing statistically significant differences between different experimental points.

### 2.7. Confocal Microscopy

Laser scanning confocal microscopy (LSCM) analysis of *N. benthamiana* tissues from inoculated plants was performed using a Zeiss 7080 Axio Observer (Zeiss, Oberkochen, Germany) equipped with a C-Apochromat 40X/1.20 W corrective water immersion objective lens. For the multicolor detection of crocins and chlorophylls, imaging was performed using the sequential channel acquisition mode. A 458 nm laser was used for excitation of crocin and chlorophyll autofluorescence, which was detected from 465 to 620 nm and from 690 to 740 nm, respectively. Images were processed using the FIJI software (Dresden, Germany), “http://fiji.sc/Fiji” (accessed on 4 April 2021).

## 3. Results and Discussion

### 3.1. Selection of Edited Lines as Chassis for the Production of Crocins

**Figure 2:**
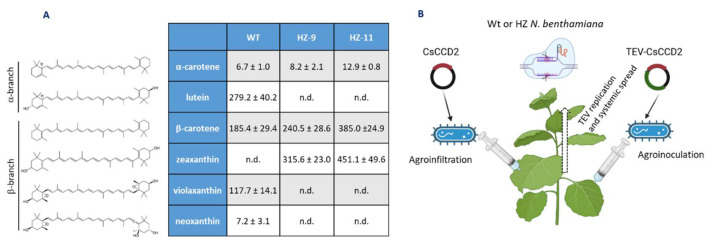
Production of saffron crocins in genome-edited *N. benthamiana*. (**A**) Carotenoid composition (µg g^−1^ dry weight (DW); average values ± standard deviation of three biological replicates) of Wt, HZ-9 (*lcye1lcye2ZEP1zep2*) and HZ-11 (*lcye1lcye2zep1zep2*) *N. benthamiana* lines (nd = not detectable); carotenoid structures are shown on the left. (**B**) Approaches used to overexpress *CsCCD2* in Wt or HZ *N. benthamiana* plants.

The LAB strain of *N. benthamiana* [18] and two previously constructed, high zeaxanthin (HZ) genome-edited lines, in the same genetic background, were utilized. Briefly, two homologous *Lycopene ε-cyclase* (*LCYe1* and *LCYe2*) and two *Zeaxanthin epoxidase* (*ZEP1* and *ZEP2*) genes present in the *N. benthamiana* allotetraploid genome were targeted. Two different edited lines were obtained: HZ-9 (*lcye1lcye2ZEP1zep2*) in which only one of the two *ZEP* genes was inactivated, and HZ-11 (*lcye1lcye2zep1zep2*) in which both *ZEP* genes were inactivated (Sulli et al., unpublished). In both lines, ε-branch (lutein) and β-branch (violaxanthin and neoxanthin) xanthophylls were completely replaced by zeaxanthin, which is undetectable in Wt lines (Figure 2A). Zeaxanthin levels were higher in the HZ-11 line, which exhibits a more stunted phenotype, probably due to a more severe deficiency in abscisic acid biosynthesis (Figure S2). These lines were used as chassis for crocin production, through the overexpression of CsCCD2 using two transient approaches: agroinfiltration using the pBI:121 vector [20] and virus-based expression using TEV [14] (Figure 2B). The latter, based on a viral vector able to replicate and move systemically through the plant, was expected to achieve larger CsCCD2 accumulation [14,37].

### 3.2. Agroinfiltration

**Figure 3:**
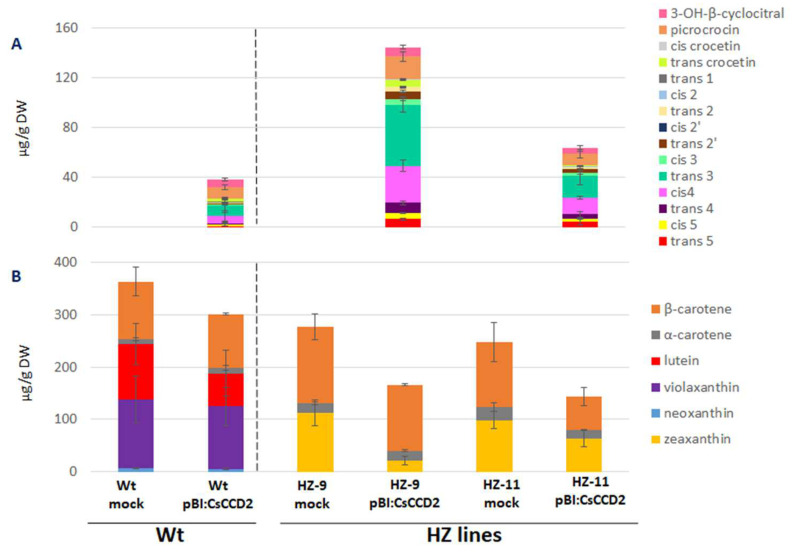
Apocarotenoid (**A**) and carotenoid (**B**) levels in Wt and HZ *N. benthamiana* plants, agroinfiltrated with pBI121 (mock) and pBI121:CsCCD2. Average values with standard deviations of three independent experiments are presented. Quantitative data can be found in Tables S1 and S2.

Leaves of Wt and HZ lines were co-infiltrated with *A. tumefaciens* harboring either pBI121 or pBI121:CsCCD2, and with another strain harboring the RK19 silencing suppressor [7]. Upon visual inspection, agroinfiltration of HZ-9 leaves was comparable to that of Wt leaves, while HZ-11 leaves proved much more difficult to agroinfiltrate, possibly due to a thickening of the leaf cuticle as a consequence of altered ABA levels (Sulli et al., unpublished). At 5 dpi, several novel apocarotenoids, attributable to the action of CCD2, accumulated in Wt leaves agroinfiltrated with CsCCD2: OH-β-cyclocitral, picrococin, crocetins, and crocins. Total apocarotenoids accumulated to 38.4 µg g^−1^ DW and crocins to 20.3 µg g^−1^ DW (Figure 3A, Table S1).

A strong increase in total apocarotenoid and crocin levels (147.2 and 115.3 µg g^−1^ DW, respectively) was observed in HZ-9 leaves, which contain higher levels of zeaxanthin, the CsCCD2 preferred substrate. In HZ-11 leaves, in spite of the higher levels of zeaxanthin, much lower total apocarotenoid and crocin levels were observed (63.4 and 48.9 µg g^−1^ DW, respectively), probably due to the less efficient agroinfiltration process. The relative abundance of different crocin isomers in leaves of Wt, HZ-9, and HZ-11 plants was similar, with *trans*-crocin 3 being the most abundant isomer, followed by *cis*-crocin 4. In addition, in the HZ lines we observed an increase in the accumulation of hyperglucosylated crocins, *trans* and *cis*-crocin 5, compared to the Wt.

The levels of zeaxanthin in HZ-9 and HZ-11 leaves were reduced upon *CsCCD2* overexpression (Figure 3B, Table S2). β-carotene levels were somewhat reduced in HZ-11, but not HZ-9 leaves. In Wt leaves, which contain undetectable levels of zeaxanthin, lutein was the major carotenoid showing significant reduction upon *CsCCD2* overexpression. These results are compatible with the fact that zeaxanthin and lutein differ only by the position of a double bond in one of the two ionone rings and with our previous results [3], showing that both zeaxanthin and lutein were substrates for CsCCD2 in vitro, yielding respectively 3-OH-β-apo-8′-carotenal and 3-OH-ε-apo-8′-carotenal, both of which are possible crocetin dialdehyde precursors.

### 3.3. Viral Expression

**Figure 4:**
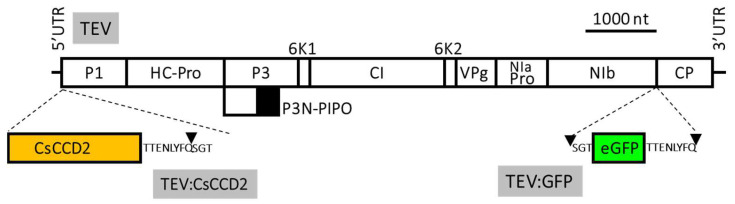
Schematic representation of wild-type TEV and recombinant clones TEV:CsCCD2 and TEV:GFP. CsCCD2 and eGFP cDNAs are represented by orange and green boxes, respectively. Lines represent TEV 5′ and 3′ UTR and boxes represent P1, HC-Pro, P3, P3N-PIPO, 6K1, CI, 6K2, VPg, NIaPro, Nib, and CP cistrons, as indicated. The sequences of the artificial NIaPro cleavage sites to mediate the release of the recombinant proteins from the viral polyprotein are also indicated. The black triangle indicates the exact cleavage site. Scale bar corresponds to 1000 nt.

We also investigated *CsCCD2* overexpression using a TEV vector [14] as a second means to overexpress *CsCCD2* in leaves of Wt and HZ *N. benthamiana* lines. Previous experiments expressing *CsCCD2* in *N. benthamiana* plants used a TEV vector with a deletion in the viral nuclear inclusion b (NIb) cistron (TEVΔNIb) to increase cargo capacity [14]. Therefore, the resulting viral recombinant clone only replicated and moved in plants in which the NIb was supplied in *trans*, such as in a transformed *N. benthamiana* line in which this viral protein was expressed under the control of cauliflower mosaic virus 35S promoter and terminator [14]. To circumvent this requirement, in this work we built a new recombinant clone based on full-length TEV, able to replicate and move in *N. benthamiana* genotypes not carrying the 35S:NIb transgene. In this vector, *CsCCD2* is expressed as the first gene product of the viral polyprotein, since this position in the viral polyprotein is crucial to efficiently target the chloroplast [23] (Figure 4). Two additional vectors, carrying the GFP (Figure 4), and an empty vector, were used as controls. The three vectors are respectively named TEV:CsCCD2, TEV:GFP, and TEV for simplicity.

**Figure 5:**
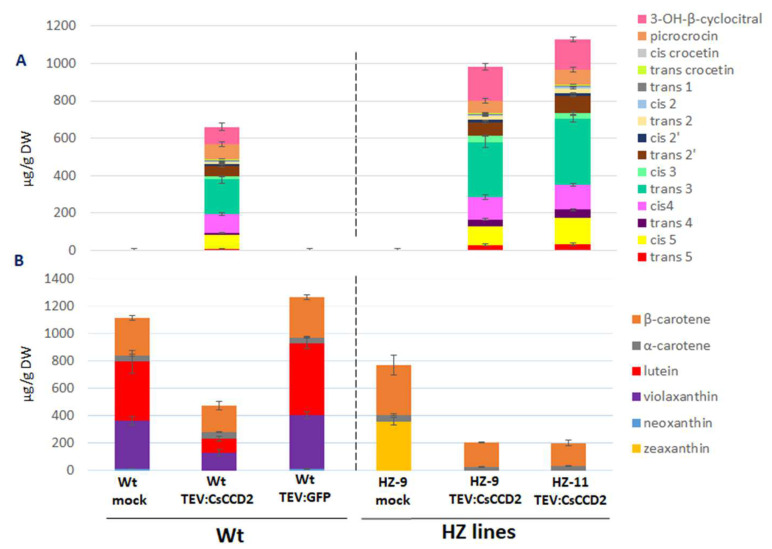
Apocarotenoid (**A**) and carotenoid (**B**) levels in Wt and HZ *N. benthamiana* plants, inoculated with TEV, TEV:GFP, and TEV:CsCCD2. Average values with standard deviations of three independent experiments are presented. Quantitative data are shown in Tables S3 and S4.

Inoculation of Wt plants with TEV:CsCCD2 yielded much higher apocarotenoid and crocin levels (660.1 and 483.1 µg g^−1^ DW, respectively) compared to agroinfiltration (Figure 5A, Table S3). These levels were further boosted in leaves of the HZ-9 and HZ-11 lines, with the latter showing the highest levels (1129.7 and 883.7 µg g^−1^ DW, respectively). Also in this case, picrocrocin and OH-β-cyclocitral accumulated at higher levels both HZ-9 lines compared to the Wt, and comparable levels were detected between the two edited lines (Figure 5A, Table S3).

Zeaxanthin was reduced to undetectable levels in both lines, while β-carotene showed a 51–53% reduction upon TEV:CsCCD2 inoculation, depending on the inoculated line (Figure 5B, Table S4). This indicates that zeaxanthin is the preferred in planta substrate for CsCCD2 and that, contrary to agroinfiltration, virus-based expression functions equally well on HZ-9 and HZ-11 lines, likely because self-replication and systemic spread of the virus overcomes the mechanical or physiological obstacles preventing efficient agroinfiltration of the latter line.

In Wt plants, where zeaxanthin is undetectable, a strong reduction in lutein, neoxanthin, and violaxanthin was observed upon TEV:CsCCD2 inoculation, suggesting that, in the absence of zeaxanthin, the latter xanthophylls become CsCCD2 substrates. Again, β-carotene showed lower (29%) reduction with respect to xanthophylls (Figure 5B, Table S4).

Taken together, these data suggest the following scenario: (i) zeaxanthin is the preferred substrate for CsCCD2 cleavage in *N. benthamiana* leaves, followed by lutein, neoxanthin, violaxanthin, and β-carotene; (ii) the higher expression levels obtained via TEV relax the observed substrate specificity of CsCCD2, making it able to cleave neoxanthin and violaxanthin, two xanthophylls that are not cleaved upon agroinfiltration; (iii) the highest crocin levels are reached in HZ-11 plants, indicating that genome editing and a virus-based expression system play additive roles in achieving maximal crocin production.

The different amenabilities of the different carotenoids to cleavage by CsCCD2 in leaves are probably due to their different structures (Figure 2), but their different localizations in the photosynthetic protein-pigment complexes could also play a role: while xanthophylls such as lutein, violaxanthin, zeaxanthin, and neoxanthin are localized in the peripheral light-harvesting complexes, β-carotene is localized exclusively in photosystem II, and preferentially in photosystem I reaction centers, where it could be less accessible to CsCCD2 cleavage [38,39].

### 3.4. Endogenous Factors and Their Contribution to Crocin Accumulation

**Figure 6:**
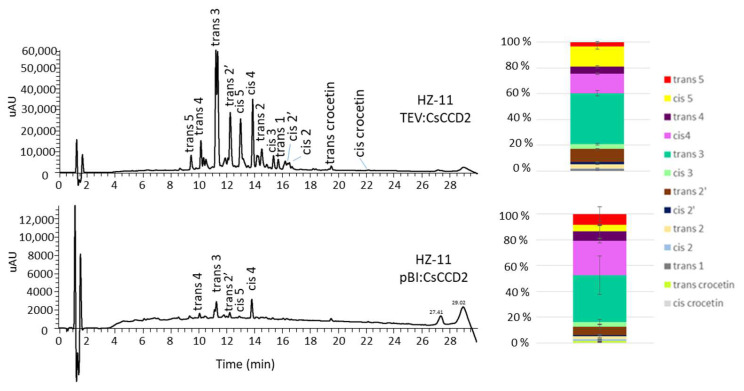
Crocin accumulation in leaves of the HZ-11 line expressing the CsCCD2 enzyme by a viral vector (TEV:CsCCD2) or agroinfiltration (pBI:CsCCD2). Quantitative data are shown in Tables S1 and S3.

HZ leaves, in which CsCCD2 is expressed either by simple agroinfiltration or using a viral vector, not only show quantitative differences in crocin accumulation, but also qualitative differences in the types of apocarotenoids accumulated (Figure 6, Tables S1 and S3). Agroinfiltrated leaves show a higher fraction of highly glycosylated crocins (48% crocins 4 and 5) than those that were virus-inoculated (40%). This is an indirect indication that the higher apocarotenoid levels achieved by the viral vector may saturate the endogenous ALDH and UGT activities present in *N. benthamiana* leaves.

**Figure 7:**
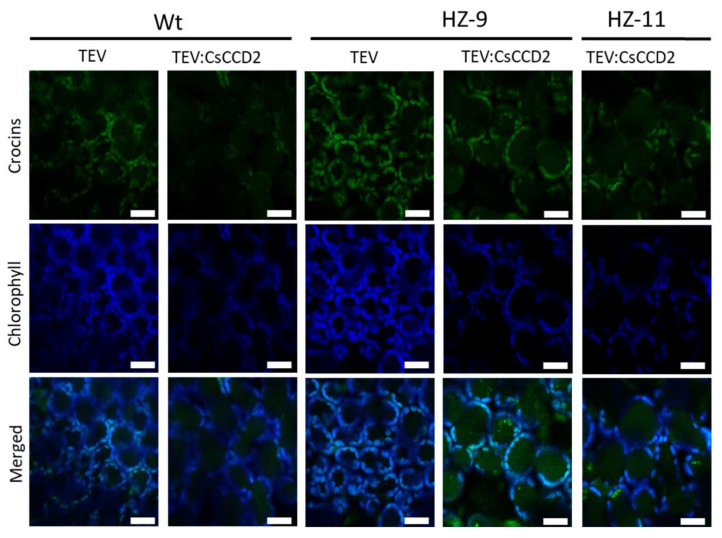
LSCM images of crocins (green) and chlorophyll autofluorescence (blue) from symptomatic Wt and HZ leaves, infected with TEV or TEV:CsCCD2, at 12 dpi. Scale bars indicate 20 µm.

Another factor contributing to accumulation of crocins is their sequestration into vacuoles [7,40]. Co-agroinfiltration of the CsABCC4a transporter in *N. benthamiana* leaves together with CsCCD2 increases the levels of crocins, indicating that endogenous vacuolar transport in *N. benthamiana* leaves may be rate-limiting for crocin accumulation [7]. Qualitative evaluation of vacuole-localized crocin autofluorescence in Wt, HZ-9, and HZ-11 leaves infected with TEV:CsCCD2 by LSCM indicates that vacuole-localized crocin fluorescence is stronger in HZ than in Wt leaves (Figure 7). We hypothesize that, as demonstrated in the case of other vacuolar transporters [41], the endogenous *N. benthamiana* transporters mediating vacuolar localization of crocins are induced by the increased abundance of their substrate. This hypothesis could be tested by transcriptomic analyses of TEV:CsCCD2–infected plants in comparison with an empty vector control.

## 4. Conclusions

*N. benthamiana* is a promising bio-factory system for the production of biopharmaceuticals and small molecules, and new plant-breeding techniques such as genome editing, agroinfiltration, and the use of viral vectors are powerful tools to deploy its full potential [42]. In this work, we demonstrated that a combination of methodologies can be used to further boost the production of saffron apocarotenoids, mainly crocins, in *N. benthamiana* leaves, as well as to study the in planta substrate specificities of carotenoid cleavage enzymes.

## Data Availability

Primary data are available in Supplementary Tables S1–S4.

## Supplementary Materials

The following supporting information can be downloaded at: https://www.mdpi.com/article/10.3390/metabo13060729/s1, Figure S1: Nucleotide sequences of wild-type TEV (sequence variant DQ986288 with two silent mutations G273A and A1119G, in red) and the derived recombinant viruses TEV-CsCCD2L and TEV-eGFP; Figure S2: Representative wt and HZ edited plants used in this study, 8 weeks after seed sowing; Table S1: Apocarotenoid accumulation (µg g^−1^ DW) in different mock and pBI:CsCCD2 agroinfiltrated lines; Table S2: Carotenoid composition (µg g^−1^ DW) of different mock and agroinfiltrated lines; Table S3: Apocarotenoid accumulation (µg g^−1^ DW) in different mock and TEV-inoculated lines; Table S4: Carotenoid composition (µg g^−1^ DW) of different mock and TEV-inoculated lines.
