# Supplementary material for: Production of Saffron Apocarotenoids in Nicotiana benthamiana Plants Genome-Edited to Accumulate Zeaxanthin Precursor
Source: Metabolites. 2023 Jun 6;13(6):729. doi: 10.3390/metabo13060729 (PMC10305034; doi:10.3390/metabo13060729)
Supplement: Supplementary file 1 [file metabolites-13-00729-s001.zip › Figure S1.pdf]

Figure S1 Nucleotide sequences of wild-type TEV (sequence variant DQ986288 with two silent mutations G273A and A1119G, in red) and the derived recombinant viruses TEV-CsCCD2L and TEV-eGFP. Cistron limits are indicated on blue background.

```
>TEV-wt (DQ986288 with two silent mutations G273A and A1119G)
AAAATAACAAATCTCAACACAACATATACAAAACAAACGAATCTCAAGCAATCAAGCATTCTACTTCTATTGCAG
CAATTTAAATCATTTCTTTTAAAGCAAAAGCAATTTTCTGAAAATTTTCACCATTTACGAACGATAGCCATGGCA
CTCATCTTTGGCACAGTCAACGCTAACATCCTGAAGGAAGTGTTCGGTGGAGCTCGTATGGCTTGCGTTACCAGC
GCACATATGGCTGGAGCGAATGGAAGCATTTTGAAGAAGGCAGAAGAAACCTCTCGTGCAATCATGCACAAACCA
GTGATCTTCGGAGAAGACTACATTACCGAGGCAGACTTGCCTTACACACCACTCCATTTAGAGGTCGATGCTGAA
ATGGAGCGGATGTATTATCTTGGTCGTCGCGCGCTCACCCATGGCAAGAGACGCAAAGTTTCTGTGAATAACAAG
AGGAACAGGAGAAGGAAAGTGGCCAAAACGTACGTGGGGCGTGATTCCATTGTTGAGAAGATTGTAGTGCCCCAC
ACCGAGAGAAAGGTTGATACCACAGCAGCAGTGGAAGACATTTGCAATGAAGCTACCACTCAACTTGTGCATAAT
AGTATGCCAAAGCGTAAGAAGCAGAAAAACTTCTTGCCCGCCACTTCACTAAGTAACGTGTATGCCCAAACTTGG
AGCATAGTGCGCAAACGCCATATGCAGGTGGAGATCATTAGCAAGAAGAGCGTCCGAGCGAGGGTCAAGAGATTT
GAGGGCTCGGTGCAATTGTTCGCAAGTGTGCGTCACATGTATGGCGAGAGGAAAAGGGTGGACTTACGTATTGAC
AACTGGCAGCAAGAGACACTTCTAGACCTTGCTAAAAGATTTAAGAATGAGAGAGTGGATCAATCGAAGCTCACT
TTTGGTTCAAGTGGCCTAGTTTTGAGGCAAGGCTCGTACGGACCTGCGCATTGGTATCGACATGGTATGTTCATT
GTACGCGGTCGGTCGGATGGGATGTTGGTGGATGCTCGTGCGAAGGTAACGTTCGCTGTTTGTCACTCAATGACA
CATTATAGCGACAAATCAATCTCTGAGGCATTCTTCATACCATACTCTAAGAAATTCTTGGAGTTGAGGCCAGAT
GGAATCTCCCATGAGTGTACAAGAGGAGTATCAGTTGAGCGGTGCGGTGAGGTGGCTGCAATCCTGACACAAGCA
CTTTCACCGTGTGGTAAGATCACATGCAAACGTTGCATGGTTGAAACACCTGACATTGTTGAGGGTGAGTCGGGA
GACAGTGTCACCAACCAAGGTAAGCTCCTAGCAATGCTGAAAGAACAGTATCCAGATTTCCCAATGGCCGAGAAA
CTACTCACAAGGTTTTTGCAACAGAAATCACTAGTAAATACAAATTTGACAGCCTGCGTGAGCGTCAAACAACTC
ATTGGTGACCGCAAACAAGCTCCATTCACACACGTACTGGCTGTCAGCGAAATTCTGTTTAAAGGCAATAAACTA
ACAGGGGCCGATCTCGAAGAGGCAAGCACACATATGCTTGAAATAGCAAGGTTCTTGAACAATCGCACTGAAAAT
ATGCGCATTGGCCACCTTGGTTCTTTCAGAAATAAAATCTCATCGAAGGCCCATGTGAATAACGCACTCATGTGT
GATAATCAACTTGATCAGAATGGGAATTTTATTTGGGGACTAAGGGGTGCACACGCAAAGAGGTTTCTTAAAGGA
TTTTTCACTGAGATTGACCCAAATGAAGGATACGATAAGTATGTTATCAGGAAACATATCAGGGGTAGCAGAAAG
CTAGCAATTGGCAATTTGATAATGTCAACTGACTTCCAGACGCTCAGGCAACAAATTCAAGGCGAAACTATTGAG
CGTAAAGAAATTGGGAATCACTGCATTTCAATGCGGAATGGTAATTACGTGTACCCATGTTGTTGTGTTACTCTT
GAAGATGGTAAGGCTCAATATTCGGATCTAAAGCATCCAACGAAGAGACATCTGGTCATTGGCAACTCTGGCGAT
TCAAAGTACCTAGACCTTCCAGTTCTCAATGAAGAGAAAATGTATATAGCTAATGAAGGTTATTGCTACATGAAC
ATTTTCTTTGCTCTACTAGTGAATGTCAAGGAAGAGGATGCAAAGGACTTCACCAAGTTTATAAGGGACACAATT
GTTCCAAAGCTTGGAGCGTGGCCAACAATGCAAGATGTTGCAACTGCATGCTACTTACTTTCCATTCTTTACCCA
GATGTCCTGAGTGCTGAATTACCCAGAATTTTGGTTGATCATGACAACAAAACAATGCATGTTTTGGATTCGTAT
GGGTCTAGAACGACAGGATACCACATGTTGAAAATGAACACAACATCCCAGCTAATTGAATTCGTTCATTCAGGT
TTGGAATCCGAAATGAAAACTTACAATGTTGGAGGGATGAACCGAGATATGGTCACACAAGGTGCAATTGAGATG
TTGATCAAGTCCATATACAAACCACATCTCATGAAGCAGTTACTTGAGGAGGAGCCATACATAATTGTCCTGGCA
ATAGTCTCCCCTTCAATTTTAATTGCCATGTACAACTCTGGAACTTTTGAGCAGGCGTTACAAATGTGGTTGCCA
AATACAATGAGGTTAGCTAACCTCGCTGCCATCTTGTCAGCCTTGGCGCAAAAGTTAACTTTGGCAGACTTGTTC
GTCCAGCAGCGTAATTTGATTAATGAGTATGCGCAGGTAATTTTGGACAATCTGATTGACGGTGTCAGGGTTAAC
CATTCGCTATCCCTAGCAATGGAAATTGTTACTATTAAGCTGGCCACCCAAGAGATGGACATGGCGTTGAGGGAA
GGTGGCTATGCTGTGACCTCTGAAAAGGTGCATGAAATGTTGGAAAAAAACTATGTAAAGGCTTTGAAGGATGCA
TGGGACGAATTAACTTGGTTGGAAAAATTCTCCGCAATCAGGCATTCAAGAAAGCTCTTGAAATTTGGGCGAAAG
CCTTTAATCATGAAAAACACCGTAGATTGCGGCGGACATATAGACTTGTCTGTGAAATCGCTTTTCAAGTTCCAC
TTGGAACTCCTGAAGGGAACCATCTCAAGAGCCGTAAATGGTGGTGCAAGAAAGGTAAGAGTAGCGAAGAATGCC
ATGACAAAAGGGGTTTTTCTCAAAATCTACAGCATGCTTCCTGACGTCTACAAGTTTATCACAGTCTCGAGTGTC
CTTTCCTTGTTGTTGACATTCTTATTTCAAATTGACTGCATGATAAGGGCACACCGAGAGGCGAAGGTTGCTGCA
CAGTTGCAGAAAGAGAGCGAGTGGGACAATATCATCAATAGAACTTTCCAGTATTCTAAGCTTGAAAATCCTATT
GGCTATCGCTCTACAGCGGAGGAAAGACTCCAATCAGAACACCCCGAGGCTTTCGAGTACTACAAGTTTTGCATT
GGAAAGGAAGACCTCGTTGAACAGGCAAAACAACCGGAGATAGCATACTTTGAAAAGATTATAGCTTTCATCACA
CTTGTATTAATGGCTTTTGACGCTGAGCGGAGTGATGGAGTGTTCAAGATACTCAATAAGTTCAAAGGAATACTG
AGCTCAACGGAGAGGGAGATCATCTACACGCAGAGTTTGGATGATTACGTTACAACCTTTGATGACAATATGACA
ATCAACCTCGAGTTGAATATGGATGAACTCCACAAGACGAGCCTTCCTGGAGTCACTTTTAAGCAATGGTGGAAC
AACCAAATCAGCCGAGGCAACGTGAAGCCACATTATAGAACTGAGGGGCACTTCATGGAGTTTACCAGAGATACT
```

GCGGCATCGGTTGCCAGCGAGATATCACACTCACCCGCAAGAGATTTTCTTGTGAGAGGTGCTGTTGGATCTGGA
AAATCCACAGGACTTCCATACCATTTATCAAAGAGAGGGAGAGTGTTAATGCTTGAGCCTACCAGACCACTCACA
GATAACGTGCACAAGCAACTGAGAAGTGAACCATTTAACTGCTTCCCAACTTTGAGGATGAGAGGGAAGTCAACT
TTTGGGTCATCACCGATTACAGTCATGACTAGTGGATTCGCTTTACACCATTTTGCACGAAACATAGCTGAGGTA
AAAACATACGATTTTGTCATAATTGATGAATGTCATGTGAATGATGCTTCTGCTATAGCGTTTAGGAATCTACTG
TTTGAACATGAATTTGAAGGAAAAGTCCTCAAAGTGTCAGCCACACCACCAGGTAGAGAAGTTGAATTCACAACT
CAGTTTCCCGTGAAACTCAAGATAGAAGAGGCTCTTAGCTTTCAGGAATTTGTAAGTTTACAAGGGACAGGTGCC
AACGCCGATGTGATTAGTTGTGGCGACAACATACTAGTATATGTTGCTAGCTACAATGATGTTGATAGTCTTGGC
AAGCTCCTTGTGCAAAAGGGATACAAAGTGTCGAAGATTGATGGAAGAACAATGAAGAGTGGAGGAACTGAAATA
ATCACTGAAGGTACTTCAGTGAAAAAGCATTTCATAGTCGCAACTAATATTATTGAGAATGGTGTAACCATTGAC
ATTGATGTAGTTGTGGATTTTGGGACTAAGGTTGTACCAGTTTTGGATGTGGACAATAGAGCGGTGCAGTACAAC
AAAACTGTGGTGAGTTATGGGGAGCGCATCCAAAGACTCGGTAGAGTTGGGCGACACAAGGAAGGAGTAGCACTT
CGAATTGGCCAAACAAATAAAACACTGGTTGAAATTCCAGAAATGGTTGCCACTGAAGCTGCCTTTCTATGCTTC
ATGTACAATTTGCCAGTGACAACACAGAGTGTTTCAACCACACTGCTGGAAAATGCCACATTATTACAAGCTAGA
ACTATGGCACAGTTTGAGCTATCATATTTTTACACAATTAATTTTGTGCGATTTGATGGTAGTATGCATCCAGTC
ATACATGACAAGCTGAAGCGCTTTAAGCTACACACTTGTGAGACATTCCTCAATAAGTTGGCGATCCCAAATAAA
GGCTTATCCTCTTGGCTTACGAGTGGAGAGTATAAGCGACTTGGTTACATAGCAGAGGATGCTGGCATAAGAATC
CCATTCGTGTGCAAAGAAATTCCAGACTCCTTGCATGAGGAAATTTGGCACATTGTAGTCGCCCATAAAGGTGAC
TCGGGTATTGGGAGGCTCACTAGCGTACAGGCAGCAAAGGTTGTTTATACTCTGCAAACGGATGTGCACTCAATT
GCGAGGACTCTAGCATGCATCAATAGACTCATAGCACATGAACAAATGAAGCAGAGTCATTTTGAAGCCGCAACT
GGGAGAGCATTTTCCTTCACAAATTACTCAATACAAAGCATATTTGACACGCTGAAAGCAAATTATGCTACAAAG
CATACGAAAGAAAATATTGCAGTGCTTCAGCAGGCAAAAGATCAATTGCTAGAGTTTTCGAACCTAGCAAAGGAT
CAAGATGTCACGGGTATCATCCAAGACTTCAATCACCTGGAAACTATCTATCTCCAATCAGATAGCGAAGTGGCT
AAGCATCTGAAGCTTAAAAGTCACTGGAATAAAAGCCAAATCACTAGGGACATCATAATAGCTTTGTCTGTGTTA
ATTGGTGGTGGATGGATGCTTGCAACGTACTTCAAGGACAAGTTCAATGAACCAGTCTATTTCCAAGGGAAGAAG
AATCAGAAGCACAAGCTTAAGATGAGAGAGGCGCGTGGGGCTAGAGGGCAATATGAGGTTGCAGCGGAGCCAGAG
GCGCTAGAACATTACTTTGGAAGCGCATATAATAACAAAGGAAAGCGCAAGGGCACCACGAGAGGAATGGGTGCA
AAGTCTCGGAAATTCATAAACATGTATGGGTTTGATCCAACTGATTTTTCATACATTAGGTTTGTGGATCCATTG
ACAGGTCACACTATTGATGAGTCCACAAACGCACCTATTGATTTAGTGCAGCATGAGTTTGGAAAGGTTAGAACA
CGCATGTTAATTGACGATGAGATAGAGCCTCAAAGTCTTAGCACCCACACCACAATCCATGCTTATTTGGTGAAT
AGTGGCACGAAGAAAGTTCTTAAGGTTGATTTAACACCACACTCGTCGCTACGTGCGAGTGAGAAATCAACAGCA
ATAATGGGATTTCCTGAAAGGGAGAATGAATTGCGTCAAACCGGCATGGCAGTGCCAGTGGCTTATGATCAATTG
CCACCAAAGAGTGAGGACTTGACGTTTGAAGGAGAAAGCTTGTTTAAGGGACCACGTGATTACAACCCGATATCG
AGCACCATTTGTCACTTGACGAATGAATCTGATGGGCACACAACATCGTTGTATGGTATTGGATTTGGTCCCTTC
ATCATTACAAACAAGCACTTGTTTAGAAGAAATAATGGAACACTGTTGGTCCAATCACTACATGGTGTATTCAAG
GTCAAGAACACCACGACTTTGCAACAACACCTCATTGATGGGAGGGACATGATAATTATTCGCATGCCTAAGGAT
TTCCCACCATTTCCTCAAAAGCTGAAATTTAGAGAGCCACAAAGGGAAGAGCGCATATGTCTTGTGACAACCAAC
TTCCAAACTAAGAGCATGTCTAGCATGGTGTCAGACACTAGTTGCACATTCCCTTCATCTGATGGCATATTCTGG
AAGCATTGGATTCAAACCAAGGATGGGCAGTGTGGCAGTCCATTAGTATCAACTAGAGATGGGTTCATTGTTGGT
ATACACTCAGCATCGAATTTCACCAACACAAACAATTATTTCACAAGCGTGCCGAAAAACTTCATGGAATTGTTG
ACAAATCAGGAGGCGCAGCAGTGGGTTAGTGGTTGGCGATTAAATGCTGACTCAGTATTGTGGGGGGGCCATAAA
GTTTTCATGAGCAAACCTGAAGAGCCTTTTCAGCCAGTTAAGGAAGCGACTCAACTCATGAGTGAATTGGTGTAC
TCGCAAGGGGAGAAGAGGAAATGGGTCGTGGAAGCACTGTCAGGGAACTTGAGGCCAGTGGCTGAGTGTCCCAGT
CAGTTAGTCACAAAGCATGTGGTTAAAGGAAAGTGTCCCCTCTTTGAGCTCTACTTGCAGTTGAATCCAGAAAAG
GAAGCATATTTTAAACCGATGATGGGAGCATATAAGCCAAGTCGACTTAATAGAGAGGCGTTCCTCAAGGACATT
CTAAAATATGCTAGTGAAATTGAGATTGGGAATGTGGATTGTGACTTGCTGGAGCTTGCAATAAGCATGCTCATC
ACAAAGCTCAAGGCGTTAGGATTCCCAACTGTGAACTACATCACTGACCCAGAGGAAATTTTTAGTGCATTGAAT
ATGAAAGCAGCTATGGGAGCACTATACAAAGGCAAGAAGAAAGAAGCTCTCAGCGAGCTCACACTAGATGAGCAG
GAGGCAATGCTCAAAGCAAGTTGCCTGCGACTGTATACGGGAAAGCTGGGAATTTGGAATGGCTCATTGAAAGCA
GAGTTGCGTCCAATTGAGAAGGTTGAAAACAACAAAACGCGAACTTTCACAGCAGCACCAATAGACACTCTTCTT
GCTGGTAAAGTTTGCGTGGATGATTTCAACAATCAATTTTATGATCTCAACATAAAGGCACCATGGACAGTTGGT
ATGACTAAGTTTTATCAGGGGTGGAATGAATTGATGGAGGCTTTACCAAGTGGGTGGGTGTATTGTGACGCTGAT
GGTTCGCAATTCGACAGTTCCTTGACTCCATTCCTCATTAATGCTGTATTGAAAGTGCGACTTGCCTTCATGGAG
GAATGGGATATTGGTGAGCAAATGCTGCGAAATTTGTACACTGAGATAGTGTATACACCAATCCTCACACCGGAT
GGTACTATCATTAAGAAGCATAAAGGCAACAATAGCGGGCAACCTTCAACAGTGGTGGACAACACACTCATGGTC
ATTATTGCAATGTTATACACATGTGAGAAGTGTGGAATCAACAAGGAAGAGATTGTGTATTACGTCAATGGCGAT
GACCTATTGATTGCCATTCACCCAGATAAAGCTGAGAGGTTGAGTGGATTCAAAGAATCTTTCGGAGAGTTGGGC
CTGAAATATGAATTTGACTGCACCACCAGGGACAAGACACAGTTGTGGTTCATGTCACACAGGGCTTTGGAGAGG
GATGGCATGTATATACCAAAGCTAGAAGAAGAAAGGATTGTTTCTATTTTGGAATGGGACAGATCCAAAGAGCCG
TCACATAGGCTTGAAGCCATCTGTGCATCAATGATCGAAGCATGGGGTTATGACAAGCTGGTTGAAGAAATCCGC
AATTTCTATGCATGGGTTTTGGAACAAGCGCCGTATTCACAGCTTGCAGAAGAAGGAAAGGCGCCATATCTGGCT

GAGACTGCGCTTAAGTTTTTGTACACATCTCAGCACGGAACAAACTCTGAGATAGAAGAGTATTTAAAAGTGTTG
TATGATTACGATATTCCAACGACTGAGAATCTTTATTTTCAGAGTGGCACTGTGGGTGCTGGTGTTGACGCTGGT
AAGAAGAAAGATCAAAAGGATGATAAAGTCGCTGAGCAGGCTTCAAAGGATAGGGATGTTAATGCTGGAACTTCA
GGAACATTCTCAGTTCCACGAATAAATGCTATGGCCACAAAACTTCAATATCCAAGGATGAGGGGAGAGGTGGTT
GTAAACTTGAATCACCTTTTAGGATACAAGCCACAGCAAATTGATTTGTCAAATGCTCGAGCCACACATGAGCAG
TTTGCCGCGTGGCATCAGGCAGTGATGACAGCCTATGGAGTGAATGAAGAGCAAATGAAAATATTGCTAAATGGA
TTTATGGTGTGGTGCATAGAAAATGGGACTTCCCCAAATTTGAACGGAACTTGGGTTATGATGGATGGTGAGGAG
CAAGTTTCATACCCGCTGAAACCAATGGTTGAAAACGCGCAGCCAACACTGAGGCAAATTATGACACACTTCAGT
GACCTGGCTGAAGCGTATATTGAGATGAGGAATAGGGAGCGACCATACATGCCTAGGTATGGTCTACAGAGAAAC
ATTACAGACATGAGTTTGTCACGCTATGCGTTCGACTTCTATGAGCTAACTTCAAAAACACCTGTTAGAGCGAGG
GAGGCGCATATGCAAATGAAAGCTGCTGCAGTACGAAACAGTGGAACTAGGTTATTTGGTCTTGATGGCAACGTG
GGTACTGCAGAGGAAGACACTGAACGGCACACAGCGCACGATGTGAACCGTAACATGCACACACTATTAGGGGTC
CGCCAGTGATAGTTTCTGCGTGTCTTTGCTTTCCGCTTTTAAGCTTATTGTAATATATATGAATAGCTATTCACA
GTGGGACTTGGTCTTGTGTTGAATGGTATCTTATATGTTTTAATATGTCTTATTAGTCTCATTACTTAGGCGAAC
GACAAAGTGAGGTCACCTCGGTCTAATTCTCCTATGTAGTGCGAGAAAAAAAAAAAAAAAAAAAAAAAAAAAAAA
AAAAAAAAAAAAAAA

>TEV-CsCCD2L (insert between possitions 144 and 145 of TEV-wt; artificial NIaPro cleavage site is in purple)
ATGGAATCTCCTGCTACTAAATTACCTGCACCTCTGCTGATGTTATCTTCTTCTCCATTCCTTCTCCCTTCTCCT
AATAAGAGCTCCTCCATCTTCCTTCCACGTAAATTAGGGCCGCTACCTCCAAAATATTATTATTACAATTGCTGC
CATCCTAAGAGTAGATCAATCTCAGTAGTATCAATGGCAAATAAGGAGGAGGCAGAGACCAGTAAGAAGAAGCCC
AAACCATTAAAAGTACTAATTACCAAAGTGGATCCGAAGCCGAGGAAGGGCATGGCATCCGTCGCAGTGGACTTA
CTCGAGAAGGCCTTTGTATACCTATTGTCCGGAAATTCTGCAGCTGATCGTAGTAGTAGTAGTGGTCGTCGTCGT
CGTAAAGAGCATTACTACCTCTCCGGCAATTATGCGCCCGTCGGACACGAAACCCCGCCCTCCGACCACCTCCCC
ATTCATGGATCCCTTCCTGAATGCTTGAATGGAGTGTTTCTGAGAGTTGGTCCTAACCCCAAGTTTGCTCCCGTA
GCCGGATACAATTGGGTCGATGGAGATGGAATGATTCATGGATTGCGTATTAAAGATGGAAAAGCAACTTATCTA
TCTCGATATATTAAAACGTCACGGTTTAAACAAGAAGAATATTTTGGAAGAGCAAAATTTATGAAGATTGGAGAT
CTAAGGGGATTGCTTGGGTTCTTTACGATCTTAATACTAGTACTTCGAACAACATTGAAAGTAATAGACATTTCA
TATGGAAGAGGGACGGGTAATACAGCTCTTGTGTATCATAATGGCTTACTATTGGCTCTATCAGAAGAAGATAAA
CCTTATGTTGTTAAAGTTTTAGAAGATGGAGACTTGCAAACTCTTGGGATATTGGATTATGACAAGAAATTGTCA
CATCCATTCACCGCTCATCCAAAGATCGATCCGTTAACTGATGAGATGTTTACCTTTGGATATTCCATCTCGCCT
CCGTATCTTACTTATCGAGTCATTTCCAAGGATGGAGTGATGCAAGATCCAGTGCAAATCTCAATTACATCCCCT
ACCATAATGCATGATTTTGCTATTACTGAAAATTATGCCATCTTCATGGACCTGCCCTTGTATTTCCAACCAGAG
GAAATGGTAAAGGGGAAATTTGTCTCTTCATTTCACCCTACAAAAAGAGCTCGTATCGGTGTGCTTCCACGATAT
GCAAAAGACGAGCATCCAATTCGATGGTTCGATCTTCCAAGTTGCTTCATGACTCATAATGCAAATGCTTGGGAA
GAGAATGATGAAGTTGTGCTATTCACATGTCGCCTTGAGAGTCCTGATCTTGACATGCTTAGTGGACCTGCGGAA
GAAGAGATTGGGAATTCAAAAAGTGAGCTTTACGAAATGAGGTTCAATTTGAAAACTGGAATTACTTCACAAAAG
CAACTATCTGTACCTAGTGTTGATTTTCCTCGGATCAACCAAAGTTATACTGGCAGGAAACAGCAATATGTTTAT
TGTACTCTTGGCAACACCAAGATTAAGGGCATTGTGAAGTTTGATCTGCAAATTGAACCAGAAGCCGGAAAGACA
ATGCTTGAAGTTGGAGGAAATGTACAAGGCATCTTTGAGTTGGGACCTAGAAGATATGGTTCAGAGGCAATATTT
GTGCCATGCCAACCTGGCATCAAATCTGATGAGGATGACGGTTACTTGATATTCTTTGTACACGACGAAAACAAT
GGGAAATCTGAGGTCAATGTCATCGATGCAAAGACAATGTCTGCAGAACCTGTGGCTGTTGTGGAACTTCCAAGC
AGGGTTCCATATGGATTCCATGCCTTGTTTCTGAATGAGGAAGAACTTCAGAAGCACCAAGCAGAGACAACAACC
GAAAATTTATATTTCCAGTCTGGAACA

>TEV-eGFP (insert between possitions 8517 and 8518 of TEV-wt; artificial NIaPro cleavage site is in purple)
TCAGGTACAATGGTGAGCAAGGGCGAGGAGCTGTTCACCGGGGTGGTGCCCATCCTGGTCGAGCTGGACGGCGAC
GTAAACGGCCACAAGTTCAGCGTGTCCGGCGAGGGCGAGGGCGATGCCACCTACGGCAAGCTGACCCTGAAGTTC
ATCTGCACCACCGGCAAGCTGCCCGTGCCCTGGCCCACCCTCGTGACCACCCTGACCTACGGCGTGCAGTGCTTC
AGCCGCTACCCCGACCACATGAAGCAGCACGACTTCTTCAAGTCCGCCATGCCCGAAGGCTACGTCCAGGAGCGC
ACCATCTTCTTCAAGGACGACGGCAACTACAAGACCCGCGCCGAGGTGAAGTTCGAGGGCGACACCCTGGTGAAC
CGCATCGAGCTGAAGGGCATCGACTTCAAGGAGGACGGCAACATCCTGGGGCACAAGCTGGAGTACAACTACAAC
AGCCACAACGTCTATATCATGGCCGACAAGCAGAAGAACGGCATCAAGGTGAACTTCAAGATCCGCCACAACATC
GAGGACGGCAGCGTGCAGCTCGCCGACCACTACCAGCAGAACACCCCCATCGGCGACGGCCCCGTGCTGCTGCCC
GACAACCACTACCTGAGCACCCAGTCCGCCCTGAGCAAAGACCCCAACGAGAAGCGCGATCACATGGTCCTGCTG
GAGTTCGTGACCGCCGCCGGGATCACTCTCGGCATGGACGAGCTGTACAAGACTACAGAGAACCTCTACTTTCAA
