# Supplementary material for: Production of Saffron Apocarotenoids in Nicotiana benthamiana Plants Genome-Edited to Accumulate Zeaxanthin Precursor
Source: Metabolites. 2023 Jun 6;13(6):729. doi: 10.3390/metabo13060729 (PMC10305034; doi:10.3390/metabo13060729)
Supplement: Supplementary file 1 [file metabolites-13-00729-s001.zip › Figure S2.pdf]

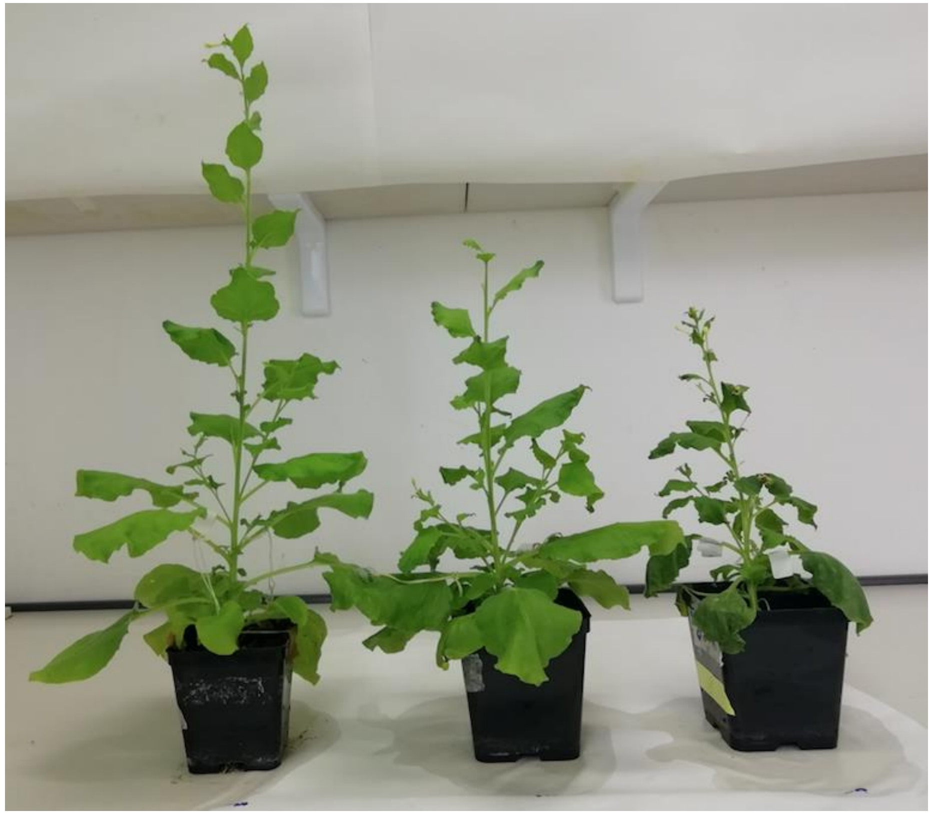

Figure S2. Representative wt and HZ edited plants used in this study, 8 weeks after seed sowing.
